# High-Sensitivity, Quantified, Linear and Mediator-Free Resonator-Based Microwave Biosensor for Glucose Detection

**DOI:** 10.3390/s20144024

**Published:** 2020-07-20

**Authors:** Alok Kumar, Cong Wang, Fan-Yi Meng, Zhong-Liang Zhou, Meng Zhao, Guo-Feng Yan, Eun-Seong Kim, Nam-Young Kim

**Affiliations:** 1 School of Information and Communication, Harbin Institute of Technology, Harbin 150001, China; kalok2705@gmail.com (A.K.); blade@hit.edu.cn (F.-Y.M.); zhouzhongliang@hit.edu.cn (Z.-L.Z.); 2 School of Mathematics and Physics, Suzhou University of Science and Technology, Suzhou 215009, China; mzhao@usts.edu.cn; 3 Research Center for Smart Sensing, Zhejiang Lab, Hangzhou 310000, China; yanguofeng@zhejianglab.com; 4 Department of Electronic Engineering, Kwangwoon University, Seoul 01897, Korea; esk@kw.ac.kr

**Keywords:** microwave biosensor, electric field, filling factor, glucose detection

## Abstract

This article presents a high-sensitivity, quantified, linear, and mediator-free resonator-based microwave biosensor for glucose sensing application. The proposed biosensor comprises an air-bridge-type asymmetrical differential inductor (*L*) and a center-loaded circular finger-based inter-digital capacitor (*C*) fabricated on Gallium Arsenide (GaAs) substrate using advanced micro-fabrication technology. The intertwined asymmetrical differential inductor is used to achieve a high inductance value with a suitable Q-factor, and the centralized inter-digital capacitor is introduced to generate an intensified electric field. The designed microwave sensor is optimized to operate at a low resonating frequency that increases the electric field penetration depth and interaction area in the glucose sample. The microwave biosensor is tested with different glucose concentrations (0.3–5 mg/ml), under different ambient temperatures (10–50 °C). The involvement of advanced micro-fabrication technology effectively miniaturized the microwave biosensor (0.006λ_0_ × 0.005λ_0_) and enhanced its filling factor. The proposed microwave biosensor demonstrates a high sensitivity of 117.5 MHz/mgmL^-1^ with a linear response (*r^2^* = 0.9987), good amplitude variation of 0.49 dB/mgmL^-1^ with a linear response (*r^2^* = 0.9954), and maximum reproducibility of 0.78% at 2 mg/mL. Additionally, mathematical modelling was performed to estimate the dielectric value of the frequency-dependent glucose sample. The measured and analyzed results indicate that the proposed biosensor is suitable for real-time blood glucose detection measurements.

## 1. Introduction

Diabetes mellitus is one of the severe life-threatening diseases. It is a kind of metabolic disorder that occurs due to variation in blood glucose beyond the normal range. This disease can affect almost every organ of the body through complications such as kidney failure, heart failure, paralysis attack, and bli [ndness, among others [1,2,3]. According to the World Health Organization (WHO) survey, approximately 422 million people had diabetes in 2017, which will increase to 592 million in 2035 [4,5]. Medically, diabetes is classified into three categories as gestational diabetes, type-1 diabetes, and type-II diabetes. According to the report of the International Diabetes Federation (IDF), around 90% of people have type-II diabetes, which occurs due to the under-utilization of hormone insulin [6]. Beyond normal range, the blood sugar level is categorized into two categories as hyperglycemia (glucose concentration > 1.20 mg/mL) and hypoglycemia (glucose concentration < 0.80 mg/mL) [7]. Thus, it is vital to examine the glucose level for both categories. The complications due to the fluctuation of blood glucose can be avoided by closely and accurately monitoring the blood glucose level through accurate biosensors [8]. Based on the sensing principle, several types of biosensors are available such as an optical type, a capacitive type, a piezoelectric type, an electrochemical type, and a microwave type [9,10,11,12,13]. 

Microwave-type biosensors are widely preferred for biomedical applications due to their high sensitivity, real-time measurement, fast response, robustness, label-free detection, and low cost [14,15,16,17]. Microwave glucose-sensing is based on the interaction of glucose samples with the time-varying electromagnetic field, thus varying the amplitude and frequency of the incident electromagnetic (EM) wave [18,19,20]. Ebrahimi et al. [21] demonstrated a complementary split-ring resonator (CSRR)-type glucose sensor to detect glucose in water solution. The sensitivity of the device was evaluated with resonance frequency shift and amplitude shift. Moreover, Lee et al. [22] investigated a reusable coplanar waveguide (CPW) device for glucose detection in aqueous solution. They also concluded that the increase in glucose concentration decreases the penetration depth of the device and increases the conductive losses. Kumari et al. [23] presented an epsilon negative (ENZ) resonator having two complementary geometries of ring and horn shape for glucose sample characterization. They discussed that the perturbation of the fringing electric field induces the shift in resonating frequency. Moreover, Kim et al. [12] proposed an integrated passive device-based microwave resonator for glucose detection in aqueous solution. The authors used 0.25 to 5 mg/mL glucose/aqueous solution concentrations to verify the high and low glucose detection limit of the proposed sensor. The authors concluded that the proposed integrated passive device-based microwave resonator was reusable with the proper clinical procedure. Similarly, Hassan et al. [24] validated an LC tank resonator for continuous detection of glucose samples in the long-range 0 to 5 mg/mL in an aqueous solution. The sensitivity of the proposed sensor was also measured with human serum albumin (HSA), which indicated a shift in resonating frequency according to HSA concentration.

Recently, researchers are using microwave sensing technology for blood glucose detection at the clinical level. Liu et al. [25] proposed a microwave resonator-based glucose sensor based on the whispering gallery mode resonance technique for in vivo detection. The authors observed that the in vivo measurement was correlated with the ex vivo measurement (glucose/water solution). Moreover, Yee et al. [26] investigated a defective ground structure for the characterization of a blood glucose sample. The authors concluded that the obtained results were consistent with the readings of a standard glucometer for blood glucose detection. The design of microwave-based glucose sensors is focused on developing a device with high reliability, small size, low limit of detection (LOD), and fast response [27,28,29]. High reliability helps to obtain precise results, and a lower LOD is essential for label-free glucose detection [30]. 

The previous studies reveal that the product of the filling factor and loaded quality(Q)-factor is essential to get high sensitivity [31,32]. Several resonators with high Q-value measurements have been demonstrated for glucose-sensing applications [33,34]. The high value of the loaded Q-factor enhances the localized electric field intensity that increases sensitivity. Unfortunately, the small values of the loaded Q-factor depend upon the material dielectric losses, parasitic losses, and conductor losses that occur due to the sample under test (SUT). Disturbance in an intensified electric field intensity can be achieved by reducing the resonating frequency and attaining a high penetration depth [35,36,37]. Additionally, the enhanced filling factor (i.e., the volume fraction of deposited dielectric sample on the sensing area) can be achieved by a fabricated structure using micro-fabrication techniques [38,39]. Moreover, human blood plasma constitutes 92% of water by the volume fraction [40]. Hence, the measurement of ambient temperature variation is critical to introduce a temperature-compensated microwave biosensor. 

In the current study, a highly sensitive, quantified, reliable, linear, and mediator-free glucose biosensor has been demonstrated. The proposed biosensor is decorated with additional features such as high penetration depth in a dielectric medium, temperature compensator, lab-on-chip compatibility, and reusable. The compact microwave biosensor (0.006λ_0_ × 0.005λ_0_) is fabricated on Gallium Arsenide (GaAs) substrate using an advanced micro-fabrication technique with minimum fabrication errors to achieve a high filling factor. The advanced structure-based intertwined air-bridge-type differential spiral inductor (L) is used to intensify the localized EM field in a circular finger-type interdigital capacitor (C). The optimization of the microwave biosensor structure is performed to achieve a low resonating frequency and high penetration depth. The proposed microwave sensor is tested with a glucose concentration of 0.3–5 mg/mL. The ambient temperature variation is shown to observe the environmental effect on the proposed microwave biosensor. The deflection of resonating frequency is succeeded within five seconds after the deposition of the tested sample, as presented in Video S1. The fast detection of the glucose sample is suitable for real-time measurements [41]. 

## 2. Materials and Methods

### 2.1. Proposed Microwave Biosensor Design 

**Figure 1:**
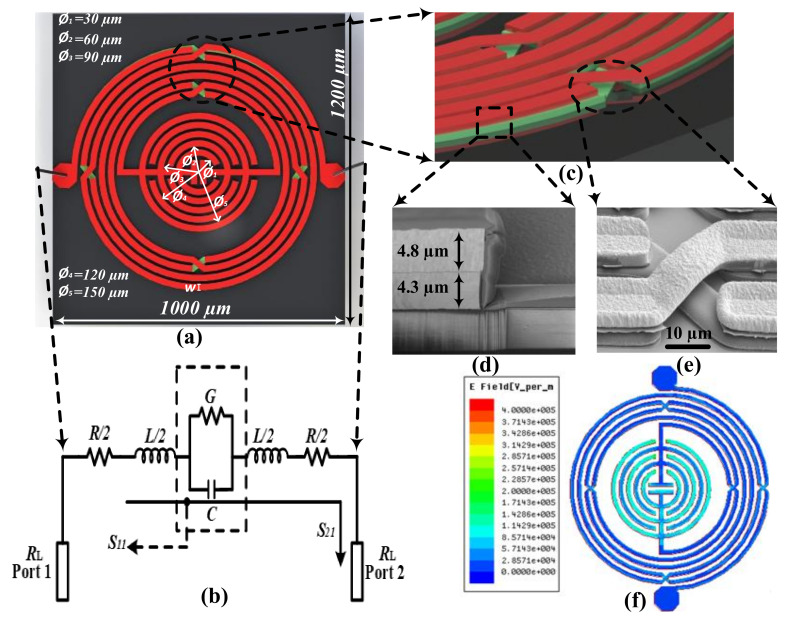
(**a**) The layout of micro-fabricated microwave biosensor, (**b**) equivalent circuit of the resonator-based microwave biosensor, (**c**) magnified view of the air-bridge structure, (**d**) scanning electron microscope (SEM) image of the air-bridge structure, focused ion beam (FIB) image of micro-fabricated microwave resonator, (**e**) top view of micro-fabricated microwave biosensor, and (**f**) E-field intensity simulation of proposed microwave biosensor indicating maximum electric field concentrated at the center of the microwave biosensor.

The proposed LC-resonator based microwave biosensor for glucose sensing is fabricated using an intertwined air-bridge-type asymmetrical differential spiral inductor and circular finger-type inter-digital capacitor. The intertwined air-bridge based asymmetrical differential spiral inductor is introduced to achieve high mutual inductance, and the circular finger-type inter-digital capacitor is used to attain a concentrated electric field. The device geometry, with its equivalent circuit, is shown in Figure 1a,b. The resonating frequency $\left(f_{r}\right)$ of the proposed microwave biosensor depends upon the inductance (*L*) and capacitance (*C*) of the structure, and can be expressed as [42]

(1) $$f_{r}=\frac{1}{2\times \pi \times \sqrt{L\times C}}$$

The overall device inductance is the combination of self-inductance induced in the single metal segment, and mutual inductance produced between adjacent metal segments can be estimated as [43]:

(2) $$L_{s}=2l_{ms}\left(\ln\frac{2l_{ms}}{W_{eff\;}+h_{ms}}-0.5\right)$$

where $l_{ms}$ represents the length of the metal segment, $h_{ms}$ represents the height of the metal segment, and $W_{eff\;}$ represents the effective (frequency-dependent) line width of the metal segment, which can be approximated as [43]

(3) $$W_{eff}=W_{0-n}\left(1-e^{\left(-\frac{W}{W_{0-n}}\right)}\right)$$

(4) $$W_{0-i}=C_{1}\times C_{2}^{n-1}\sqrt{\frac{1}{f}}$$

where n represents the number of turns in the inductor coil, W represents the physical width of the metal segment, $C_{1}$ and $C_{2}$ represent linearized parameters for resistance and inductance, respectively, and f represents the resonating frequency. The effect of mutual inductance is vital to consider for a miniaturized resonator as the mutual inductance depends on the gap (*g*) between adjacent segment tracks and can be represented as [43]

(5) $$\pm L_{mut}=2\times 10^{-4}l_{ms}\left(\ln\left(\frac{l_{ms}}{g}+\sqrt{1+\frac{l_{ms}^{2}}{g^{2}}}\right)-\;\sqrt{1+\frac{l_{ms}^{2}}{g^{2}}}+\frac{g}{l_{ms}}\right)$$

The sign of mutual inductance depends on the direction of current flow, positive for in-phase currents, and negative for out-phase currents. Equations (3) and (5) above indicate that self-inductance mainly depends on the metal segment’s length, whereas mutual inductance depends on the metal segment’s length and the gap between the adjacent metal electrode. In the proposed structure, an intertwined air-bridge-type differential spiral inductor is designed to enhance the overall device inductance. The magnified image of the fabricated two metal layers and air-bridge structure of the asymmetrical differential spiral inductor is shown in Figure 1d,e, respectively.

Similarly, the capacitance value is another factor that affects the resonating frequency of a microwave device. The device capacitance of ($C_{d}$) can be expressed in terms of substrate permittivity $\varepsilon_{GA}$, and circular finger length $L_{cf}$ [44]:

(6) $$C_{d}=\left[\varepsilon_{0}\varepsilon_{GA\;}\left(\frac{1+\varepsilon_{GA\;}\;}{2}\right)\frac{K\left(\sqrt{1+k^{2}}\right)}{K\left(k\right)}+\varepsilon_{0}\frac{h_{ms}}{W_{eff}}+{\left(\frac{K\left(k\right)}{\varepsilon_{0}K\left(\sqrt{1+k^{2}}\right)}\right)}^{-1}\right]\left(n-1\right)L_{cf}$$

(7) $$\;\;k=\left(1+\frac{2\times W}{2\times g+W}\right)\times \left(\sqrt{\frac{1}{1+\frac{2\times W}{g}}}\right)$$

(8) $$K=\sqrt{1-k^{2}}$$

where $K\left(k\right)$ and k=wg represent the first elliptical integrals.

**Figure 2:**
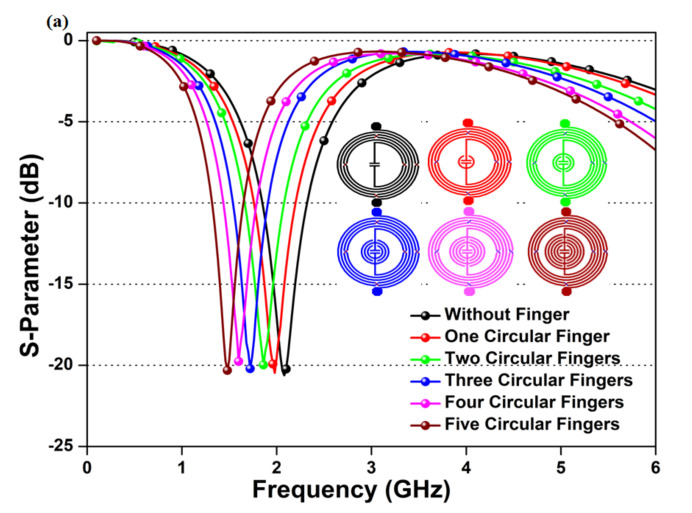
Optimization of the proposed microwave resonator on the number of circular fingers, the inset image represents micro-fabricated devices with different circular fingers.

In the designed microwave biosensor, the S parameter depends on the inductive coupling that exists between the intertwined air-bridge-type asymmetrical differential spiral inductor lines and capacitive coupling between the circular finger-type inter-digital capacitor lines. The concentrated electric field intensity and penetration depth are two vital factors in achieving high sensitivity while preserving a low sample volume [45,46]. Figure 1f illustrates the High-Frequency Structure Simulator (HFSS)-simulated concentrated electric field intensity at the resonating frequency. The presented image shows that the electric field is highly concentrated at the circular fingers of the inter-digital capacitor, indicating that the capacitive effect is more potent than the inductive effect in the proposed glucose-based microwave biosensor. Moreover, the penetration depth is another vital factor that affects the sensitivity of microwave biosensors. A high penetration depth allows the generated EM wave to interact with all ions present in the tested sample. Practically, the high penetration of the EM wave heats the target sample and excites the electrons from the valence band to the conduction band [35]. The induced EM wave polarizes the conduction band electrons and generates a resonating frequency shift. The penetration depth $\left(D_{p}\right)\;$ can be calculated using the below equation [46]:

(9) $$D_{p}=\frac{c}{2\times \pi \times f_{r}\times {\left(2\varepsilon_{sam}\right)}^{0.5}}{\left[{\left(1+{\left(tan\delta \right)}^{2}\right)}^{0.5}-1\right]}^{-0.5}$$

where c represents the speed of the electric wave in free space, $\varepsilon_{sam}$ represents a dielectric constant of the deposited sample on the sensing area, and tanδ represents the loss factor of the deposited sample on the sensing area. The penetration depth is affected by the dielectric property of the deposited sample and the resonating frequency of the device. Hence, the designed microwave biosensor is operated at a low center frequency of 1.50 GHz to attain deeper penetration and broad-area interaction. The calculated penetration depth of the proposed microwave biosensor towards a DI water sample having $\varepsilon_{sam}$ = 80.52 and tanδ = 0.073 is 34.46 mm. Moreover, the value of the inductor and the capacitor is optimized to achieve a low center frequency with a low-profile design. The proposed microwave biosensor is designed with an intertwined air-bridge-type asymmetrical differential spiral inductor to a high inductance value with a suitable Q-factor [47]. Further, the inscribed circular finger-type inter-digital capacitor is optimized to generate high capacitance and achieve a low resonating frequency, as shown in Figure 2. Each circular finger increases the device capacitance, thus decreases the resonating frequency of the microwave biosensor, as represented in Equation (1). 

### 2.2. Advanced Micro-Fabrication of Proposed Microwave Biosensor

The proposed band-pass filter-type microwave biosensor is fabricated using an advanced micro-fabrication technique. The used advanced fabrication technology offers high-yield, low-loss, miniaturized, and cost-effective microwave devices [48]. In this fabrication process, GaAs is used as a substrate on which an intertwined air-bridge-type asymmetrical differential spiral inductor and circular finger-type inter-digital capacitor are engraved. The proposed LC-resonator based microwave sensor is fabricated on a 6-inch GaAs substrate. In a single fabrication, the 6-inch GaAs substrate allows to fabricate 20,000 microwave sensor devices of the same size. After the calculation of all possible expenditures, the estimated cost of the proposed sensor in mass fabrication is around 1 RMB/sensor.

The detailed fabrication process is isolated in two parts: first, the fabrication of the first metal layer, and second, the formation of the second metal layer and air-bridge post for the asymmetrical differential spiral inductor, explained as [49]:

- Formation of first metal layer: First, the GaAs substrate was cleaned carefully with acetone, iso-propyl alcohol, and distilled water, respectively. The wet cleaning helps to reduce the surface defects and improve surface roughness to enhance the layer’s adhesion. Afterward, the SiN_x_ layer of 0.2 µm thickness was deposited on the GaAs substrate as a wafer passivation layer through a plasma-enhanced chemical vapor deposition (PECVD) process. The first seed metal (Ti/Au) was sputtered with the thickness of (0.02 µm /0.08 µm) on the SiN_x_ layer to increase the adhesion between the first metal layer and GaAs substrate. Subsequently, the photoresistor (PR) was patterned on the GaAs substrate to engrave the first metal layer with the desired structure. The first metal layer (Cu/Au) with the thickness of (4.5 µm /0.5 µm) was electroplated on the surface to design the first layer of the asymmetrical differential spiral inductor and circular finger-type inter-digital capacitor. The thicker layer of copper (4.5 µm) was preferred due to its high operational speed, better conductivity, and low cost. After that, the PR layer was stripped off through the lift-off process, and later dry etching was performed to remove the unwanted seed metal. Again, the 0.2 µm thick SiN_x_ layer was deployed on the first metal layer using the PECVD technique to prevent short-circuiting between metals layers and protect sidewalls.
- Formation of the second metal layer and air-bridge post*:* Furthermore, the PR coating was deposited on the SiN_x_ layer to craft the desired structure for the second metal layer. Afterward, reactive ion etching (RIE) was applied using a plasma etcher to remove the unwanted passivation layer. Then, PR removing was done to remove the PR layer using the lift-off process. In the next step, the PR layer was deposited to form an air-bridge post followed by a hard-baking process. Afterward, the second seed metal (Ti/Au) with the height of (0.02 µm /0.08 µm) was sputtered to enhance adhesiveness between the first metal layer and the second metal layer. Again, the PR layer is deposited to design the air-bridge metal layer. Subsequently, the second metal layer (Cu/Au) with a thickness of (4.5 µm/ 0.5 µm) is formed to create an air-bridge in the asymmetrical differential spiral inductor. Furthermore, the undesired second seed metal was removed through the RIE process, and the unwanted PR of the air-bridge post and air-bridge metal layer was etched using wet etching. In the last step, the final passivation layer of 0.3 µm was introduced to cover all fabricated component areas that protect them from environmental impurities such as dust, humidity, and scratches, among others.

### 2.3. Proposed Microwave Biosensor Fabrication and Testing

**Figure 3:**
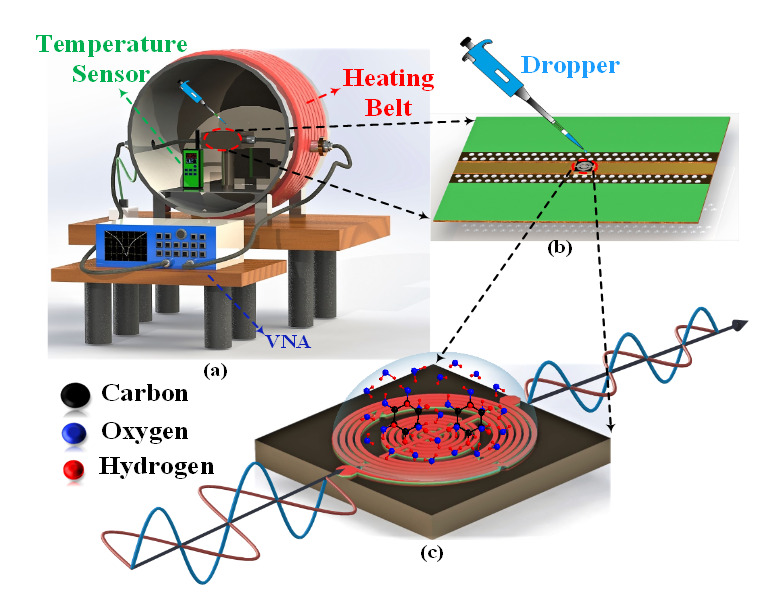
(**a**) 3D schematic of measurement setup for microwave-based glucose sensing, (**b**) 3D schematic of micro-fabricated biochip mounted printed circuit board (PCB), and (**c**) sensing mechanism of proposed GaAs-based micro-fabricated microwave biosensor. (**a**) 3D schematic of measurement setup for microwave-based glucose sensing, (**b**) 3D schematic of micro-fabricated biochip mounted printed circuit board (PCB), and (**c**) sensing mechanism of proposed GaAs-based micro-fabricated microwave biosensor.

The proposed resonator-type microwave biosensor was simulated using Keysight’s Advanced System Design (ADS) electromagnetic simulator and later fabricated on GaAs substrate using an advanced micro-fabrication process. The optimized microwave biosensor was designed using a GaAs substrate having a dielectric constant $\varepsilon_{GA}$ = 12.9, loss tangent tan δ = 0.0002, and height = 200 µm. Additionally, the air-bridge structure was formed by using two metal layers (Ti/Au) of thickness around 5 µm (single layer). The fabricated microwave biosensor was installed in the measurement chamber for the glucose sensing measurements, as shown in Figure 3a. The measurement chamber was a specially designed chamber having high-frequency wires made of polytetrafluoroethylene (PTFE), and connectors made of ceramic. Moreover, the fabricated microwave biosensor is low-profile (0.006λ_0_ × 0.005λ_0_) and cannot connect directly to the measurement system. Hence, the fabricated device first attached to the specially designed general-purpose printed circuit board (PCB) with wire bonding, as shown in Figure 3b. The wire-bonding length must be optimized precisely to eliminate the effect of parasitic inductance and capacitance. Afterward, the general-purpose PCB, along with the fabricated microwave biosensor, was installed in the measurement chamber for the S parameter measurements. The placed device was connected with Keysight Technologies’ FieldFox Handheld Vector Network Analyzer (VNA) (N9916A, Santa Rosa, CA, USA) to measure and record reflection and transmission coefficients. The heating belt was placed around the measurement chamber to test the heating effect on glucose sensing. The heating belt was connected with the external temperature sensor (XC-24-K-12, Omega, Biel/Bienne, Switzerland) to precisely control the belt temperature. The reading of the external temperature sensor was carefully selected so that the internal temperature could not fluctuate. Moreover, the internal heating measurement was measured by evaluating the internal temperature sensor (TECPEL 320) readings. The temperature sensor was placed near the testing device to get a more accurate reading for ambient temperature sensing analysis. Prior to the S parameter measurement, the inner chamber temperature near to the biosensor device is calculated carefully to get accurate measurement readings. The prepared glucose samples are dropped using a microdropper on the sensing area to get high sensitivity. 

### 2.4. Sample Preparation and Characterization

The glucose-based aqueous solution was prepared to measure the sensing performance of the proposed resonator-based microwave biosensor. The aqueous solution was prepared using a quantized mixture of D-Glucose powder (Sigma Aldrich) and deionized water (Merck Millipore, Billerica, MA). The glucose solution was calibrated using standard concentrations of 0.3, 1, 2, 3, 4, and 5 mg/mL. The glucose sample’s range (0.3–5 mg/mL) was selected to cover diabetes patients with hyperglycemia (glucose concentration > 1.20 mg/mL) as well as hypoglycemia (glucose concentration < 0.80 mg/mL) [50]. A one hundred-nanoliter (100 nL) droplet was dropped on the sensing area with a digitally variable pipette (0.1–2.5 μL), purchased from Shanghai LC-BX Instrument Technology Co Ltd., to quantify the sample volume of the glucose solution. All samples are tested at room temperature with a fixed-volume pipette to achieve a fixed volume and shape.

## 3. Result and Discussion

### 3.1. S Parameter Characterization of Glucose Solution

**Figure 4:**
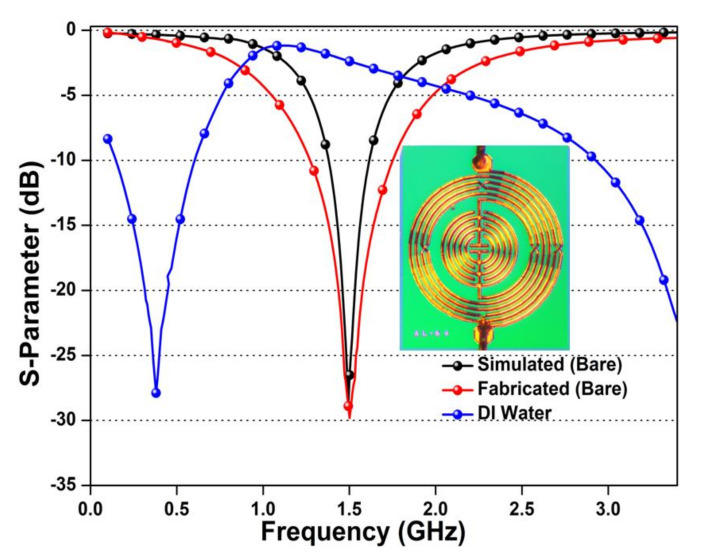
S parameter (S_11_) of simulated, measured, and DI water deposited on the micro-fabricated microwave biosensor with an inset image of a micro-fabricated microwave biosensor.

The accuracy of the advanced micro-fabrication technique is estimated by comparing the S parameter of the simulated and fabricated device. The evaluated S parameter (S_11_) of the simulated, fabricated, and dropped DI water on the sensing region is shown in Figure 4. The measured S parameter exhibited small deflection between the simulated and fabricated microwave sensor, which occurred due to the fabrication errors and connectors’ impedance mismatching. The smaller distance between adjacent metal segments generates parasitic effects at high frequencies. The effect creates a virtual inductance and capacitance in the circuit. This induced virtual inductance and capacitance generate losses in the circuit and affect the overall fabricated device results. Moreover, the inset image presents the high-quality and uniformly fabricated resonator-based microwave biosensor. The DI water droplet has high permittivity that interacts with generated EM waves of the fabricated microwave biosensor. This interaction increases the device inductance and capacitance values, thus generating a downward shift in the resonating frequency. 

**Figure 5:**
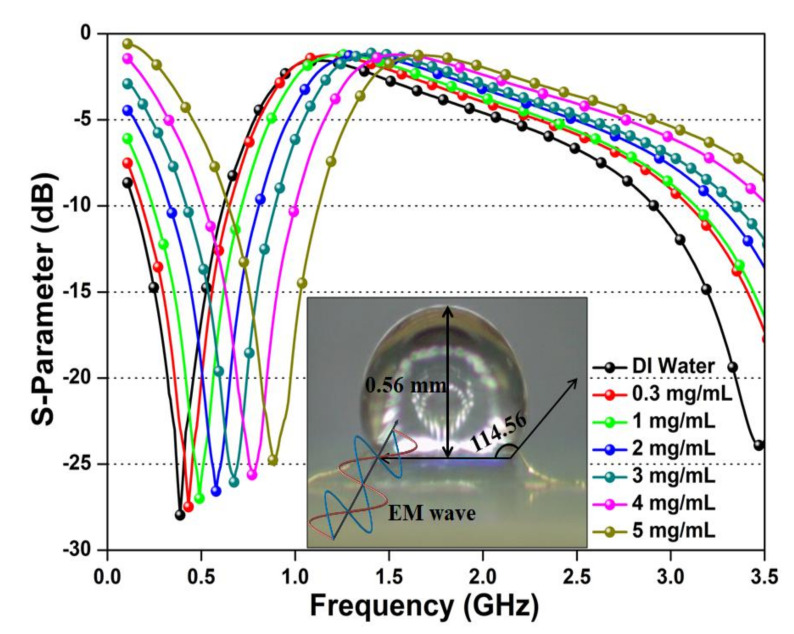
Measured center frequency shift and amplitude variation of micro-fabricated microwave biosensor after interaction of glucose–water solution with different concentrations (0.3–5 mg/mL), the inset image represents the optical microscopic image of the dropped glucose–water sample with hydrophobicity analysis located at the sensing region.

Additionally, the S parameter variation of the fabricated microwave biosensor with different glucose samples (0.3–5 mg/mL) is presented in Figure 5. The sample testing is performed at room temperature and later with ambient temperature variations. The presented results were recorded instantly (< 5 s) after the test sample dropped on the sensing region. The obtained results indicate the rise in resonating frequency with the increase in glucose concentration. The upward shift in resonating frequency is due to increased viscosity and reduces the dielectric constant of the tested glucose solution. However, the amplitude of the resonating frequency decreases as the glucose concentration is increased. The presence of amplitude declination is due to the interaction of the EM wave with glucose molecules that generate losses in the incident EM wave. Figure 5 signifies that the advanced fabrication process can be used to achieve a high filling factor. The contact angle measurement and volume analysis are also performed to classify the nature and formation of the sample droplets. The obtained contact angle of 114.56° indicates that the microwave biosensor surface has a hydrophobic nature. The hydrophobic property of the microwave biosensor helps to control the shape and size of the deposited sample precisely. Moreover, the height of the deposited drop is 0.56 mm, which is very small as compared with the penetration depth (34.46 mm) that allows the electric waves to penetrate the deposited sample and interact with each molecule; thus, the proposed microwave sensor attains high sensitivity.

### 3.2. Linearization of Derived Parameters

**Figure 6:**
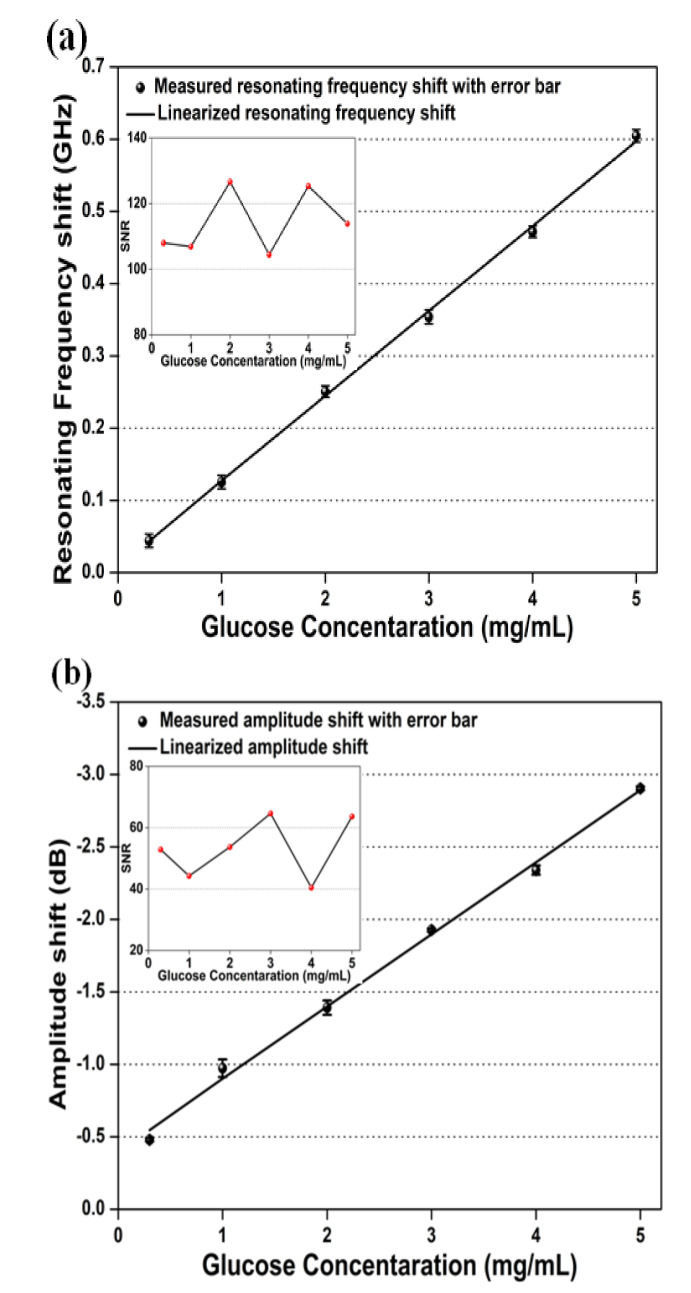
Calibration curve based on linear regression for (**a**) central frequency shift and (**b**) amplitude shift with error bars (n = 6) indicating reproducibility and inset image representing the signal to noise analysis.

In microwave biosensors, the linearization of the S parameter helps to calibrate the derived parameters after the interaction of the target sample with the induced EM wave. In the proposed microwave biosensor, linear regression was applied to analyze the resonant frequency shift and wave amplitude variation with different glucose concentrations. The applied linear regression analysis presented in Figure 6a reveals a good correlation with the linear fit of *r^2^* = 0.9987 (*r* = correlation coefficient) between the glucose concentration and resonating frequency shift. This relationship can be expressed as

(10) $$f_{shift}=0.11750\;G_{conc}+0.00815$$

where $f_{shift}$ represents the resonating frequency shift and $G_{conc}$ represents the glucose concentration (mg/mL). The above relation reveals the proposed microwave biosensor exhibits a high sensitivity of 117.50 MHz/mg mL^-1^ towards 100 nL quantized samples. The deflection in the measurement results of sextuple detection at the same glucose concentration could occur during the preparation of glucose/aqueous solution and the deposition of the sample during the measurement. The preparation errors have been attempted to be minimized by using high-precision electronic clinical weighing machines and containers. However, the occurrence of an error during sample deposition cannot be neglected. In this article, the error bars have been calculated using the RSD of the sextuple analysis of each glucose concentration. The maximum RSD of 0.78% was attained at 2 mg/mL, which indicates good reproducibility of all tested samples. The minimal deflection in the results of the frequency shift during the sextuple measurement of the same glucose concentration is due to the human error that occurred during the sample deposition. The LOD and limit of quantification (LOQ) for the resonating frequency shift were calculated as 0.35 and 1.1 mg/mL, respectively. The inset image in Figure 6a depicts the signal to noise ratio (SNR), which was estimated using the ratio of the mean and standard deviation of the sextuple resonating frequency calculated at different glucose concentrations. The maximum and minimum SNR values were extracted as 126.6 dB at 2 mg/mL and 104.4 dB at 3 mg/mL, respectively.

Similarly, Figure 6b displays the linear regression analysis applied to the variation in amplitude shift with different glucose concentrations. The obtained results indicate an excellent correlation of *r^2^* = 0.9954 between the amplitude shift and different glucose concentrations, which can be approximated as

(11) $$A_{\mathrm{shift}}=0.49800{\;G}_{\mathrm{conc}}+0.39600$$

The above equation indicates a high sensitivity of 0.498 dB/mgmL^-1^ towards 100 nL quantized samples. The error bars were estimated using the sextuple analysis of each glucose sample. The maximum RSD of 1.1% was observed at 1 mg/mL, which indicates an excellent reproducibility among all tested samples. The minimal deflection in the results of the amplitude shift during the sextuple measurement of the same glucose concentration is due to the human error that occurred during sample deposition. The LOD and LOQ for amplitude shifts were analyzed as 0.63 and 2.2 mg/mL, respectively. The inset image in Figure 6b illustrates the SNR of the sextuple amplitude variation calculated at different glucose concentrations. The maximum and minimum SNR values were estimated as 89.1 dB at 4 mg/mL and 74.8 dB at 3 mg/mL, respectively.

### 3.3. Temperature Effect 

**Figure 7:**
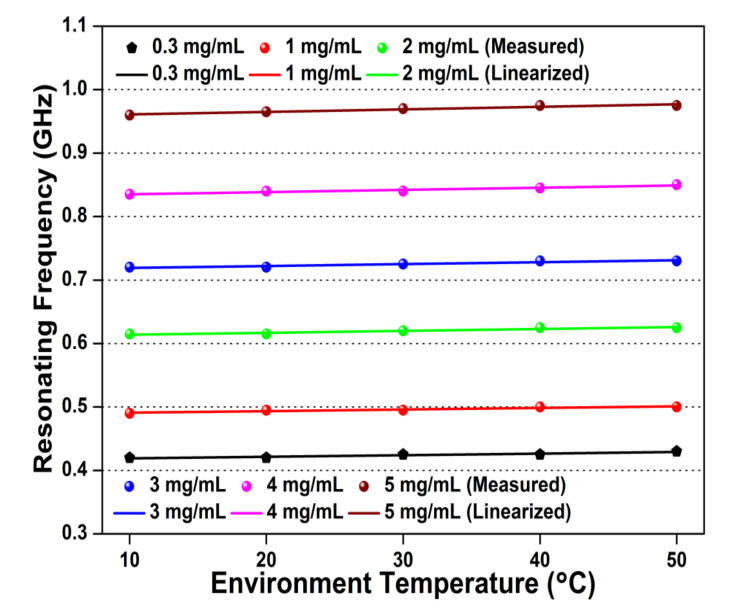
Temperature dependency of resonating frequency for different glucose concentrations (0.3–5 mg/mL).

The blood plasma organizes with 92% of water, hence the temperature effect is a critical parameter to scrutinize for glucose sensing application. In the proposed microwave biosensor, the ambient temperature is varied from 10 to 50 ℃ to estimate the effect on glucose sample testing. The temperature effect on resonating frequency for different glucose concentrations (0.3–5 mg/mL) is shown in Figure 7. The obtained result indicates a linear, unidirectional, and minute upward shift in the resonating frequencies of the proposed microwave biosensor with temperature variation. The sensitivity results of temperature-dependent variation in resonating frequency are represented as 0.00025, 0.00025. 0.00030, 0.00035, and 0.00040 GHz/℃ for the glucose sample concentrations of 0.3, 1, 2, 3, 4, and 5 mg/mL, respectively. The low-sensitivity, linear, and unidirectional response towards temperature variations demonstrates the proposed biosensor is temperature-independent. 

### 3.4. Modeling of the Microwave Biosensor 

The parametric analysis of the glucose-sensing principle in the proposed microwave biosensor was validated by approximating the relationship between change in the frequency-dependent complex effective permittivity and glucose concentration. This relationship was evaluated by creating a matrix correlation between the measured resonating frequency shifts $\;\left(\Delta f_{r}\right)$, the loaded Q-factor shift $\left(\Delta Q_{L}\right)$, and the calculated characteristic matrix, represented as [51]

(12) $$\left[\begin{array}{c} \Delta \varepsilon_{Glucose}^{’}\\ \Delta \varepsilon_{Glucose}^{“} \end{array}\right]=\left[\begin{array}{cc} -0.3713 & 5.0600\\ -0.1723 & 2.3980 \end{array}\right]\;\left[\begin{array}{c} \Delta f_{r}\\ \Delta Q_{L} \end{array}\right]$$

The ideal complex permittivity value of different glucose samples was estimated using the Debye model theory equation, expressed as [45]

(13) $$\varepsilon^{*}=\varepsilon^{\prime }+\varepsilon^{“}=\varepsilon_{\infty }+\frac{\varepsilon_{static}-\varepsilon_{\infty }}{1+i\omega \tau }\;\varepsilon_{\infty }=-8.214\times 10^{-12}\times g^{2}\left(mg/mL\right)+2.148\times 10^{-5}\times g\left(mg/mL\right)+8.722\;\varepsilon_{s}=\;\;\;2.318\times 10^{-13}\times g^{2}\left(mg/mL\right)-2.793\times 10^{-6}\times g+81.015\;\tau \left(ps\right)=-8.370\times 10^{-13}\times g^{2}\left(mg/mL\right)+5.150\times 10^{-6}\times g+8.776$$

**Figure 8:**
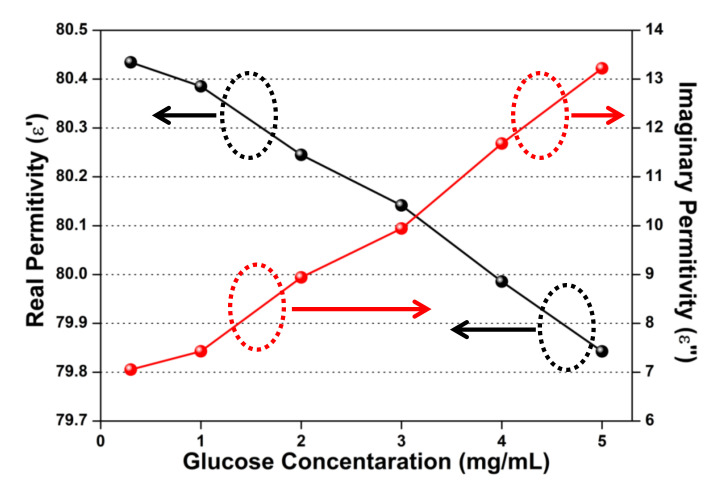
Calculated complex permittivity variation for different glucose concentrations.

The values of $\varepsilon_{\infty }$, $\varepsilon_{s}$, and $\;\tau \left(ps\right)$ in the equations were derived using a commercial dielectric probe measured with different glucose concentrations. The elements of the characteristic matrix were calculated using the complex permittivity relation presented in Equation (13). The estimated values of $\varepsilon_{Glucose}^{’}$ and $\varepsilon_{Glucose}^{“}$, derived from the measured values of the $\Delta f_{r}$ and $\Delta Q_{L}$ relations presented in Equation (12), are shown in Figure 8 and Table 1. The measured values of $\Delta f_{r}$ and $\Delta Q_{L}\;$ have been obtained at a 1.5 GHz frequency that was further used to evaluate the values of $\varepsilon_{Glucose}^{’}$ and $\varepsilon_{Glucose}^{“}$, hence the calculated permittivity values are frequency-dependent. 

The obtained results indicate that the real permittivity ($\varepsilon_{Glucose}^{’}$) is negatively correlated with glucose concentration, and imaginary permittivity ($\varepsilon_{Glucose}^{“}$) is positively correlated with glucose concentration. This correlation represents that the enhanced glucose sample concentrations induce a decrease in complex permittivity and generate high losses.

### 3.5. Sensing Mechanism of the Proposed Microwave Biosensor

The sensitivity of the microwave biosensor is related to the normalized change in resonating frequency due to the change in sample permittivity and interrupted electric field intensity. Microwave biosensor sensing can be expressed using perturbation theory represented as [42]

(14) $$\left|\frac{\Delta f_{r}}{f_{r}}\right|=\frac{\int_{v}^{v+\Delta v}\left(\left[\mu +\Delta \mu \right]{\left|H_{0}\right|}^{2}-\left[\varepsilon +\Delta \varepsilon \right]{\left|E_{0}\right|}^{2}\right)dv}{\int_{0}^{v}\left(\mu {\left|H_{0}\right|}^{2}+\varepsilon {\left|E_{0}\right|}^{2}\right)dv}$$

where v, μ, and ε represent fixed volume, unperturbed permeability, and unperturbed permittivity, respectively, $H_{0}$ and $E_{0}$ represent undisturbed magnetic and electric field (consider for simplicity), respectively, and Δv, Δμ, and Δε represent a change in volume, permeability, and permittivity of the target sample. The tested glucose samples are considered to be pure dielectric; hence, the magnetic component can be neglected. The modified Equation (14) can be expressed as

(15) $$\left|\frac{\Delta f_{r}}{f_{r}}\right|=\frac{\int_{v}^{v+\Delta v}\left(\left[\varepsilon +\Delta \varepsilon \right]{\left|E_{0}\right|}^{2}\right)dv}{\int_{0}^{v}\left(\varepsilon {\left|E_{0}\right|}^{2}\right)dv}$$

The above relation indicates the change in resonating frequency can be related to the volume of the sample, variation in effective permittivity, and induced electric field intensity. In the designed microwave biosensor, the high reproducibility in sensing results indicates the size and position of the tested samples are precisely considered. If the resonating frequency is high and the penetration depth is low, then the incident EM wave might get absorbed near the surface of the biosensor, thus producing inaccurate results [27]. In the proposed microwave biosensor, the resonating frequency is considered as low to get a high penetration depth and a broad interaction that give accurate results in each testing sample. The frequency-dependent variation with glucose concentration can be governed by the polarization of water ions in the induced electric field. The frequency-dependent variation of complex permittivity can be expressed using the Debye dispersion model, demonstrated as [44]

(16) $$\varepsilon_{Glucose-sample}=\varepsilon_{glucose-sample}^{’}+\varepsilon_{glucose-sample}^{“}=\left[\frac{\varepsilon_{static}-\varepsilon_{\infty }}{1+\omega^{2}\tau^{2}}+\varepsilon_{\infty }\right]+j\left[\frac{\left(\varepsilon_{static}-\varepsilon_{\infty }\right)\omega \tau }{1+\omega^{2}\tau^{2}}\right]$$

The above equation represents that the change in complex permittivity depends on the $\varepsilon_{static}$ (glucose sample permittivity) and τ (relaxation time) of the tested glucose concentrations. The glucose molecules (C_6_H_12_O_6_) have collectively linked OH bonds and H bonds that oppose the orientation of ions after the interaction of the electric wave with water molecules. This interaction disturbs the induced electric field and results in a decrease in the dielectric constant. Moreover, the glucose molecules have a large dipole moment (7.8) that induces sluggishness in the prepared glucose solution, resulting in an increase in relaxation time. The enhanced relaxation time decreases the overall effective permittivity of the microwave biosensor, thus increasing the resonant frequency. Additionally, the interaction of the EM wave with the glucose sample absorbs energy and reduces the amplitude of the incident wave. The 3D schematic of escalation in resonating frequency and decline in signal amplitude after the interaction of the glucose sample is shown in Figure 3c. However, the atmospheric temperature variation affects the relaxation time and the complex permittivity that results in a shift of resonating frequency. The relation between temperature and relaxation time can be estimated as

(17) τ=τ0eEτkBT

where $E_{\tau }$ represents diffusion change carries, and $k_{B}$ represents Boltzmann’s constant (1.3807 × 10^−23^ J/K). The Equations (16) and (17) indicate that the enhanced temperature escalates the relaxation time and decreases the complex effective permittivity. In low environment temperatures, the strong orientation of water molecules enhances the dielectric constant. As the temperature increases, more hydrogen ions form long hydrogen bond chains, which are hard to polarize by the electric field intensity. Additionally, the enhancement of atmospheric temperature induces nucleation of aqueous glucose samples and dwindles the dielectric constant at high temperature, thus decreasing the dielectric constant and increasing the resonating frequency [52]. The comparison of the proposed LC resonator-based microwave biosensor for glucose sensing application with previously published glucose sensors is shown in Table 2. The comparative results indicate that the tested sensor is miniaturized, requires a smaller sample concentration, and is highly sensitive in both cases (frequency shift and amplitude shift). Moreover, the proposed sensor exhibited a low limit of detection, which is suitable to detect glucose samples in aqueous solutions. 

## 4. Conclusions

In this article, a high-sensitivity, quantified, linear, and fast response LC resonator-based microwave biosensor is proposed for aqueous glucose sensing application. The advanced micro-fabrication technique is performed to achieve a high filling factor. The intertwined air-bridge-type differential spiral inductor is constructed to intensify the EM field in a circular finger-type interdigital capacitor. Moreover, the center-loaded-placed circular-type interdigital capacitor is optimized to achieve a low resonating frequency and high penetration depth. The micro-fabricated microwave biosensor is tested under different aqueous glucose concentrations from 0.3 to 5 mg/mL. The response and recovery time towards all tested samples of the fabricated microwave biosensor is less than 5 seconds for all tested samples. The temperature variation (10–50 ℃) analysis reflects that the proposed microwave biosensor is temperature-independent. Additionally, mathematical modelling was performed to estimate the complex permittivity of the frequency-dependent glucose sample. The extension of the current work is to detect the glucose sample present in human serum albumin at room ambient. The human serum albumin will be extracted from the human blood sample via the centrifugation process. The human blood will be taken out from a group of people regardless of their age, gender, and diabetic problems with proper medical care. The current studies indicate that the proposed microwave biosensor detects the glucose concentration through the interaction of the electric field intensity with glucose molecules. Hence, it is essential to extract HSA from the blood samples so that the effect of hematological blood property can be neglected. The proposed microwave biosensor is miniaturized, temperature-compensated, linearized, and reusable, which makes it suitable for real-time blood glucose detection, such as the point-of-care test (POCT) applications.

## Figures and Tables

**Table 1:** Calculated values of complex permittivity for different glucose concentrations.

	0.3 mg/mL	1 mg/mL	2mg/mL	3 mg/mL	4 mg/mL	5 mg/mL
Real Permittivity	80.434	80.385	80.244	80.141	79.985	79.842
Imaginary Permittivity	7.052	7.427	8.942	9.9433	11.679	13.221

**Table 2:** Comparison of proposed LC-resonator based microwave glucose sensor with previously published glucose sensors.

Ref.	Biosensor Structure	Concentration (mg/mL)	Size (λ_0_×λ_0_)	Sample Concentration (µL)	Sensitivity (MHz/mg/mL)	Sensitivity (dB/mg/mL)	Limit of Detection (mg/mL)
12	LC-Resonator	0.25–5	0.026 × 0.060	1	108	NA	0.8
19	CSRR Resonator	0.3–4	0.251 × 0.386	NA	NA	0.03	NA
21	CSRR Resonator	0–5	NA	70	0.5	0.5	NA
23	ENG Resonator	20–100	0.273 × 0.136	2	1.7	0.1	0.4
41	Hilbert-shaped resonator	0.5–2.5	0.408 × 0.808	500	NA	0.0156	0.192
This work	LC-Resonator	0.3–5	0.006 × 0.005	0.1	117.5	0.49	0.35

## Supplementary Materials

The following are available online at https://www.mdpi.com/1424-8220/20/14/4024/s1, Video S1: Measured response time of fabricated LC-resonator based microwave glucose sensor.
